# Essential packages of health services in low-income and lower-middle-income countries: what have we learnt?

**DOI:** 10.1136/bmjgh-2022-010724

**Published:** 2023-01-18

**Authors:** Ala Alwan, Gavin Yamey, Agnès Soucat

**Affiliations:** 1 DCP3 Country Translation Project, London School of Hygiene & Tropical Medicine, London, UK; 2 Duke Global Health Institute, Duke University, Durham, North Carolina, USA; 3 Division of Health and Social Protection, French Development Agency (AFD), Paris, France

**Keywords:** Health policies and all other topics, Health policy, Health services research, Health systems

In September 2015, all United Nations (UN) Member States adopted the Sustainable Development Goals (SDGs) as an integrated global agenda to chart a new era for development and poverty reduction.^1^ One of the key targets (SDG 3.8) is for countries to achieve universal health coverage (UHC) by 2030.^2^ Four years later, in September 2019, Heads of State and Government convened a high-level meeting at the UN General Assembly and committed to scale up efforts to accelerate progress towards SDG 3.8, adopting the most effective, evidence-based, high-impact and quality-assured interventions and using public spending as the main driver. Achieving UHC has three dimensions: increasing population coverage for all, expanding the range of health services and reducing financial risk. A key question, addressed in a new collection in the *BMJ Global Health* (box 1), is: how best can low-income and lower-middle-income countries (LLMICs) optimise the use of public funds, and design and implement affordable packages of health services to achieve the three UHC dimensions and ensure that all people have access to the health care they need?

Box 1 **The seven papers in the collection**

Paper 1. Alwan *et al*. Country readiness and prerequisites for successful design and transition to implementation of essential packages of health services: Experience from six countries.^5^

Paper 2. Baltussen *et al*. Decision-making processes for essential packages of health services: experience from six countries.^6^

Paper 3. Gaudin *et al*. Using costing to facilitate policy making toward Universal Health Coverage: findings and recommendations from country-level experiences.^7^

Paper 4. Soucat *et al*. From Universal Health Coverage health services packages to budget appropriation: the long journey to implementation.^8^

Paper 5. Reynolds *et al*. Building implementable packages for universal health coverage.^9^

Paper 6. Siddiqi *et al*. The role of the private sector in delivering essential packages of health services: lessons from country experiences.^10^

Paper 7. Danforth *et al*. Monitoring and evaluating the implementation of essential packages of health services.^11^

One strategic framework used by LLMICs to identify the services to be prioritised for public subsidy is the evidence provided and the approach adopted by the third edition of the Disease Control Priorities (DCP3) and its model service packages. DCP3 provides a systematic review of the evidence, including cost-effectiveness, of a wide range of health services to support policymakers in decision-making on the highest impact investments in the context of limited resources. Based on the DCP3 evidence, two generic model UHC packages of essential health services were launched in December 2017 as a starting point for evidence-informed country-specific analysis of priorities that LLMICs can consider in designing their packages and charting the roadmap to 2030.^3^ The essential UHC package includes 218 health sector interventions for lower-middle-income countries and a subset of these are distilled into a ‘highest priority package’ of 108 interventions recommended for low-income countries. In addition to its focus on investing in high-priority interventions, DCP3 also addresses the three UHC dimensions. A properly designed package of essential health interventions, funded publicly or through prepayment schemes, will reach all people, improve access to these services and reduce financial risk.

The demand for technical assistance to LLMICs in UHC-related public policies is growing, and an increasing number of countries have been using the DCP3 evidence and approach to develop and implement their own essential packages of health services (EPHS). Some of these countries have been technically supported by the DCP3 Country Translation Project, based at the London School of Hygiene & Tropical Medicine and funded by the Bill & Melinda Gates Foundation.^4^ Besides the provision of technical support, the project is also reviewing the experience of these countries, with the aim of extracting lessons learnt and updating technical guidance for other countries. A network of 60 experts has been involved in reviewing the experience of six countries in developing their national packages: Afghanistan, Ethiopia, Pakistan, Somalia, Sudan and Zanzibar-Tanzania. The review process has involved seven groups of professionals addressing specific areas of EPHS development and three review meetings organised in Geneva and London between September 2021 and March 2022. The collaboration resulted in the seven papers published in this collection (box 1).

Achieving UHC in 8 years from now is challenging for most countries, but it is even more complex in LLMICs. There are major health system gaps in all countries and policymakers often struggle in their efforts to address the gaps in know-how and in capacity to address them. A solid grasp of the barriers impeding progress is essential. By building on existing knowledge and experience on priority setting and design and implementation of UHC packages, we believe the review and analysis of country experience published in this collection provide important messages and lessons learnt that other countries can consider.

It is clear from country experience that designing essential packages of health services does not always contribute to UHC policies and programmes. Countries have little to gain if there is no transition from package development to roll-out. In paper 1, Alwan *et al* 5 use country experience to review barriers to package design and transition to implementation and highlight certain prerequisites that determine a successful outcome. For example, high-level political commitment translated into concrete actions is paramount; setting or revising an EPHS must be led, executed and owned by countries. Early and meaningful engagement of all relevant stakeholders, especially the planning and finance government sectors, is essential. Affordability and health system strengthening are critical for the transition to implementation. There is limited value in investing in package development without a realistic financing plan along the timeline for reaching UHC targets. Aspirational packages and those developed with inadequate engagement of national authorities are less likely to be implemented. Finally, sustainability for implementing UHC packages requires leadership, political stability, sustained resources and institutionalisation of technical and managerial capacity within Ministries of Health and partner institutions.

Priority setting is central to package development in the context of UHC. In paper 2, Baltussen *et al* 6 describe a stepwise approach for prioritisation of health services. The way countries organise their decision-making processes in developing or revising their EPHS can have far-reaching consequences for the scope, content, fairness and impact of the EPHS. All six countries included in the review followed a similar stepwise approach in their decision-making process. Nevertheless, they organised the specific steps and choice of decision criteria differently. Here again, the authors advise countries to prioritise stakeholder involvement, which is key to fostering fairness in decision making. For sustained impact, countries should institutionalise their decision-making process, through a legal framework, to ensure ongoing EPHS revision.

Costing is important to ensure that such packages go beyond aspiration—they should be feasible for a country to implement within its budget. In paper 3, Gaudin *et al* 7 reviewed how five of the six countries estimated the cost of their EPHS and found wide variation in the way costing methodologies were implemented. According to the analysis presented in this paper, the variation was particularly stark with respect to common health systems-related costs, methodologies used, capacity constraints and the lack of integration between costing and budgeting. They recommend building long-term institutional capacity in costing for better reliability and policy relevance. Costing and budgeting should be integrated, and EPHS costing should be linked to budget cycles.

There are usually high expectations for what an EPHS can achieve for health financing. In particular, some policymakers hope that the packages will lead to an increase in public resources. However, Soucat *et al* 8 in paper 4 argue that using EPHS to directly leverage funds for health has rarely been effective—though it can provide the basis for pooling funds. The authors also note that EPHS can translate indirectly into increased revenue through fiscal measures, and that the development and revisions of EPHS are essential to core strategic purchasing activities. Ultimately, packages need to translate into adequate public financing appropriations through country health programme design.

Reynolds *et al* 9 highlight, in paper 5, areas that are important in building implementable packages. Key elements of package design, structure and content, they argue, can affect the chances of successful implementation. As is also stressed by Alwan *et al*,^5^ the failure to incorporate delivery considerations already at the prioritisation and design stage can result in packages that undermine feasibility of implementation and the goals that countries have for service delivery. In contrast, a well-designed package can support a country in bridging effectively from prioritisation to implementation.

EPHS are mostly being delivered by the public sector. However, Siddiqi *et al* 10 argue in paper 6 that the role of the private health sector, which too often remains untapped, is essential for package design and implementation. Many LLMICs have mixed health systems, with an extensive and heterogeneous private health sector and varying degrees of governance effectiveness. In such countries, it is not realistic—at least in the short term—to provide EPHS using the public sector alone. Nevertheless, there remain important unanswered questions about engaging the private sector in implementing EPHS, including questions of accountability, quality, efficiency and governance.

Paper 7 of the supplement focuses on monitoring and evaluation (M&E) of EPHS in the context of UHC.^11^ EPHS development and implementation processes have historically paid little attention to M&E efforts. Danforth *et al* in paper 7 believe there is a lack of empirical, country-derived precedent on how to conceptualise and execute M&E activities around EPHS-related reforms. M&E plans need to be integrated into the UHC policy process right from the start and these plans should be aligned with the global monitoring framework for UHC and national health information system structures and processes building from the SDG 3.8.1 and 3.8.2 indicators on service coverage and catastrophic expenditures, respectively. The M&E framework should include a combination of these two global indicators and with a set of dynamic, country-specific indicators that assess EPHS implementation along the timeline of the SDG3.8 target.

This review and similar experiences in countries striving to achieve UHC are providing many lessons learnt that LLMICs can consider in accelerating progress to meet the 2030 SDG targets. To address the learnings from such experiences, policymakers in LLMICs will require much stronger technical support than what is currently provided.^12^ There is a pressing need to reinforce technical cooperation in these countries in priority setting, health technology assessment, and in the development and implementation of affordable UHC packages of essential health services. This calls for establishing a sustainable and viable strategy with a robust technical support platform to deliver on the UHC commitments. The development of such a strategy requires the commitment and active engagement of WHO, World Bank, other relevant multilateral agencies, experienced and motivated technical institutions and development partners.

## Data Availability

Data sharing not applicable as no datasets generated and/or analysed in this editorial.
